# Impact of intracellular radionuclide distribution in a Monte Carlo biophysical 3D multi‐cellular model for targeted alpha therapy

**DOI:** 10.1002/mp.17917

**Published:** 2025-07-15

**Authors:** Victor Levrague, Mario Alcocer‐Ávila, Sarah Leilla Otmani, Lydia Maigne, Etienne Testa, Michaël Beuve, Rachel Delorme

**Affiliations:** ^1^ Université Grenoble Alpes, CNRS, Grenoble INP, LPSC‐IN2P3 Grenoble France; ^2^ Université de Lyon, Université Claude Bernard Lyon 1, CNRS/IN2P3, IP2I Lyon, UMR5822 Villeurbanne France; ^3^ Université Clermont Auvergne, CNRS/IN2P3, LPC Clermont‐Ferrand France

**Keywords:** biophysical modeling, intracellular radionuclide distribution, microdosimetry, Monte Carlo simulations, targeted alpha therapy, tumor control probability

## Abstract

**Background:**

To understand and predict the therapeutic efficacy of targeted alpha therapy (TAT), nano‐ and microdosimetry are needed to consider the very heterogeneous dose deposition at cellular and subcellular levels.

**Purpose:**

The objective of this study is to theoretically evaluate the importance of cell internalization of alpha‐emitters on relevant dosimetric and biological endpoints.

**Methods:**

Isolated cells and realistic 3D multi‐cellular geometries (spheroids modeled with CPOP) were generated as well as distributions of alpha‐emitters corresponding to various cellular internalization cases. The alpha particles emitted were tracked with Geant4 (Monte Carlo) simulations. We calculated mean specific energies deposited into each cell nucleus ($Z_{n}$), cell survival fractions using the NanOx biophysical model, values of relative biological effectiveness (RBE) and tumor control probabilities (TCP) for each scenarios. The impact of spheroid compaction and size, alpha particle energy and radionuclide daughter diffusion was studied. The impact of the heterogeneous distribution of a number of alpha particles per cell was also studied, using a lognormal probability law.

**Results:**

For a given activity per cell (APC), the radionuclide distribution had a critical influence on $Z_{n}$ in isolated cancerous cells or small spheroids (<50 μm radius), while its impact was relatively low in larger and more compact spheroids, with a maximum variation of 30% between the distributions. For an average 10% cell survival, RBE was found to be approximately between 2.3 and 3.3, depending on the spatial radionuclide distribution and the activity distribution per cell. TCP of 1 was always obtained with an APC larger than 0.534 mBq when a uniform tumoral distribution of radionuclides was considered, and for APC larger than 0.801 mBq with a lognormal distribution. However, below these activities, TCP could strongly depend on the radionuclide distributions up to a factor of 9.5 with a uniform distribution and 1.5 for a lognormal one.

**Conclusions:**

According to these findings, a precise modeling of alpha‐emitter intracellular distributions may be required for small micro‐metastases or tumors presenting regions with relatively low radionuclide concentration in order to limit the prediction uncertainties on biological outputs. Intratumoral fluctuations of APC were also found to be a critical parameter to consider for therapeutic efficacy prediction in TAT.

## 1 INTRODUCTION

Targeted alpha therapy (TAT) has seen growing interest over the last years, with the improvements made in the development of new radiopharmaceuticals.^1^ The potential of alpha particles in radiotherapy is high, but several challenges still need to be overcome to optimize and deliver TAT treatments safely, which requires interdisciplinary field knowledge. Being able to accurately predict the radiation dose to the target region and healthy organs could guide the optimal prescribed activity and improve patient outcomes, as it was shown in internal radiation therapy using beta‐emitters.^2^ However, TAT dosimetry presents additional challenges, notably due to the high biological effects of alpha particles and their highly heterogeneous dose deposition at the micrometer level, due to the small path length of alpha particles (50–90 μm). Providing tools to predict more precisely the biological effects of TAT, according to different irradiation hypotheses, may help to guide preclinical and clinical trials in the future.

Previous in silico studies have shown that the effect of cell internalization is important to consider in molecular radiotherapy using Auger‐ or alpha‐emitters. For example, the study by Falzone et al.^3^ demonstrated the importance of internalizing Auger‐emitters in cell nuclei to gain therapeutic efficacy. It also showed that the calculation of biological parameters such as tumor control probability (TCP), instead of absorbed dose only, is more relevant to address clinical recommendations. Besides, Guerra Liberal et al.^4^ performed a quantitative intracellular impact study with alpha‐emitters, in a mono‐cellular model, with the TOPAS Monte Carlo (MC) code.^5^ The absorbed dose to the cell nucleus increased by more than a factor of 7 when the alpha particle source was in the nucleus only rather than on the membrane. Few studies have been performed to quantify the effect of radionuclide distribution in multi‐cellular models for TAT,^6^, ^7^, ^8^, ^9^, ^10^ showing that the impact on mean cell nucleus absorbed dose or cell survival was lower compared to the mono‐cellular case. For example, Oliveira‐Silva et al.^10^ calculated S‐values in mono‐ and grid‐arranged multi‐cellular models for different intracellular radionuclide distributions. They quantified the influence of nucleus source compared to cytoplasm only, as well as the progeny migration, for various alpha‐emitters. Our study aims to extend the quantification of these effects systematically, up to the computation of TCP.

Intratumoral fluctuations of radionuclide distribution may also have a major role in treatment outcome. Lognormal distributions of the number of radionuclides per cell were observed in in vitro experiments in the literature.^11^, ^12^, ^13^, ^14^ This type of distribution can dramatically change the mean survival in multi‐cellular irradiations. For example, in the in vitro study by Pasternack et al.,^13^ the distribution of several antibodies labeled in cells was measured and found to be lognormal‐like. Cell survivals were calculated based on these measurements with a MC program, and were compared with an assumed uniform antibody distribution, which led to a difference up to $10^{16}$ in the cell surviving fraction (in the case of 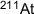 with specific activity of $4.25.10^{15}$ Bq/mol and an antibody cocktail concentration in the medium of 1 μg/mL). In addition, considering radionuclide daughter diffusion seems crucial for accurate dose estimates, as discussed in the work of Tronchin et al.^15^ A major impact was shown in a monocellular study using 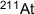,^16^ but it was not yet quantified in multi‐cellular geometries.

The objective of the present work is to quantify, in 3D multi‐cellular geometries, the importance of intracellular distribution of alpha‐emitters according to a large panel of irradiation parameters that may add uncertainties in TAT treatment predictions. This includes spheroid size and compaction, alpha‐emitter (with associated alpha energies), daughter diffusion, and cell line. The impact of using a lognormal instead of a uniform intratumoral activity (ITA) distribution was also investigated. We used detailed Geant4 MC simulations^17^, ^18^, ^19^ coupled with realistic multi‐cellular geometries,^20^ and a modified version of the NanOx biophysical model,^21^ that has been adapted to low‐energy ions for its application to TAT, to calculate relevant biological endpoints such as TCP.

The tools developed in this work must be seen as fundamental research aiming at understanding the main parameters of influence for potential clinical predictions, including the impact of uncertainties when micro‐heterogeneity is unknown.

## 2 MATERIALS AND METHODS

### 2.1 Monte Carlo calculations and physical indexes

**FIGURE 1:**
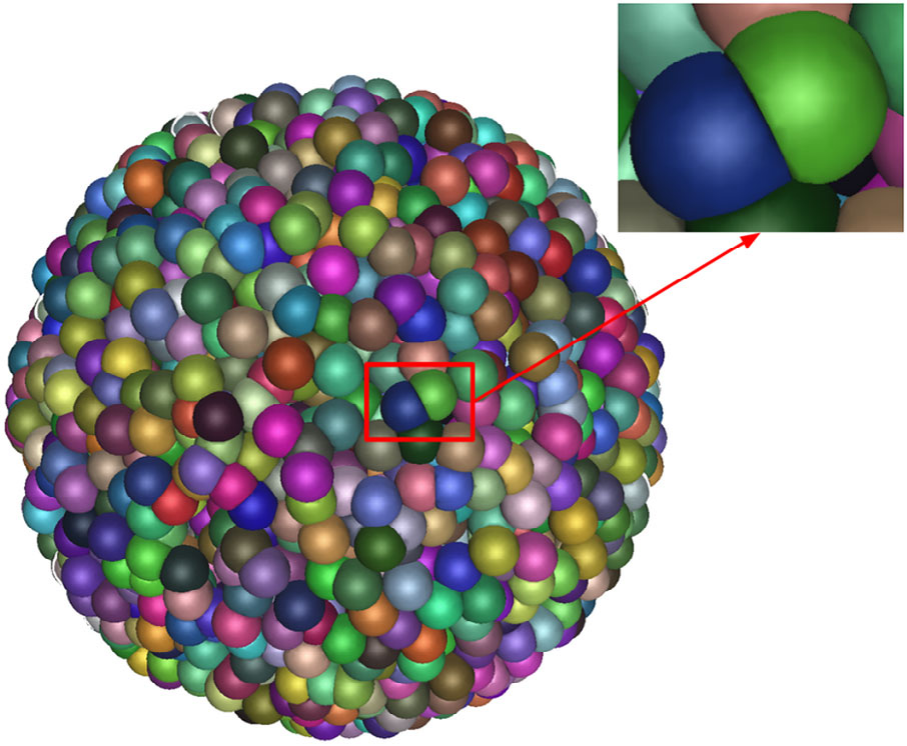
Spheroid of 95 μm radius with 75% spheroid compaction (3069 cells), as generated by CPOP^20^ (these parameters correspond to the default irradiation parameters along with the use of the 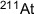 radionuclide). The zoomed inlet corresponds to two cells with truncated cytoplasm by the CPOP compaction algorithm.

The CPOP open‐source software^20^ was used to model realistic multi‐cellular geometries. This code can generate highly compacted spheroids allowing cell deformation, as illustrated in Figure 1. CPOP is coupled with the Geant4 MC simulation tool, allowing the tracking of particles in spheroids to collect data at organelle, cell, and cell population levels for dosimetry and biophysical calculation purposes. We modified CPOP to adapt the code for TAT applications, in a new CPOP 2.0 version available on GitHub.^22^

Irradiation configurations were produced with a random generation of the alpha‐emitter position in cells for a given accumulated activity in the spheroid. Alpha‐emitters (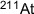, 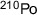, and 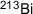) are generated uniformly in the requested cell compartment (membrane, cytoplasm, and/or nucleus), and alpha particles are emitted isotropically. It is also possible in CPOP to define multiple spheroid regions with different activities, but we considered the whole spheroid as the only region for the purpose of the present study. The simulated emission spectra were limited to the complete alpha emission rays and their secondary electrons (using the database from the Decay Data Evaluation Project international collaboration^23^) without considering beta and gamma emissions in the dose and biophysical calculations.^24^ This is justified because their impact in this intracellular distribution study would be constant due to their high range compared to alpha particles and spheroid size. For example, photons emitted by alpha‐emitters travel centimeters of water before being entirely attenuated. For each irradiation configuration, the initial ($E_{i}$) and final ($E_{f}$) kinetic energies of each alpha particle crossing a cell nucleus were collected at the entrance and exit of this nucleus, respectively. Furthermore, the total dose absorbed by the spheroid, as well as the mean specific energy, $Z_{n}$, deposited in the cell nuclei were registered, cumulated on the full irradiation configuration. Specific energy is a dose at the microscopic level, corresponding to the quotient of energy imparted by ionizing radiation to the mass of the medium.^25^ The cross‐fire contribution, $C_{n}$, to the nucleus specific energy was computed. It represents the proportion of specific energy in a cell coming from sources external to this cell and was calculated as:
(1)

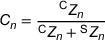

where 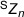 is the mean self‐specific energy to the nucleus, that is due to particles emitted from this cell, and 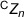, the cross‐specific energy, is the one coming from particles emitted from other cells.

The mean specific energy $Z_{n}$ and the cross‐fire contribution $C_{n}$ are the physical indexes of interest in this work.

We used Geant4 version 10.05 and the “G4EmStandardPhysics option4” *physics list* to produce the presented results. According to the literature,^19^, ^26^ this *physics list* includes the most precise models for low‐energy ions among standard Geant4 models. However, as Geant4‐DNA *physics lists* ^27^, ^28^ are more detailed for energies below 1 MeV/n, comparisons were made between “G4EmStandardPhysics option 4” and “Geant4‐DNA option 2” *physics lists* to quantify the potential impact of *physics list* choice.

When considering 10 MeV alpha particles (113 μm path length according to the NIST ASTAR database^29^), we found a 4.3 ± 0.6 μm range difference in water (+3.8%) between the *physics lists*, with the higher range for the Geant4‐DNA one. To quantify this range difference impact on our TAT simulation configurations, we considered a 95 μm radius spheroid irradiation with a 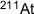 irradiation of 42 alpha particles per cell (corresponding to the experimental condition of Chouin et al.^30^) and found a 1.4% difference on the mean specific energy per cell (14 Gy ± 0.2 Gy) between *physics lists*. These differences were considered negligible, as expected. Given that the calculations performed with the Geant4‐DNA *physics list* were longer by five orders of magnitude, “G4EmStandardPhysics option4” was chosen for the rest of the study. The maximum step length was set to 0.1 μm.

### 2.2 Biophysical calculations and indexes

With low‐energy alpha particles, the absorbed dose to nuclei is not sufficient to predict biological effects. Cell survival probabilities were calculated with the NanOx biophysical model that is described in detail elsewhere.^21^, ^31^ The full cell survival modeling method is composed of, first, MC track structure simulations^32^, ^33^ for physics and radiochemistry modeling. Second, micro‐ and nanodosimetric quantities are computed to consider the stochasticity of energy deposits in critical targets. Finally, NanOx itself produces mono‐energetic radiobiological outputs that can be used with another MC simulation tool (e.g., Geant4 in this work) to compute cell survivals. NanOx was previously validated in the context of hadrontherapy by comparing cell survivals to mono‐energetic ion beam irradiations, including He ions, on a wide energy range and for 3 cell lines (Human salivary gland HSG, Chinese hamster lung fibroblast V79, and Chinese hamster ovary CHO‐K1).^34^ The total lethal damage events caused in a cell by a particle track is computed as a combination of local lethal damage, intended to reflect the production of complex, irreparable, DNA damage in nanometric targets, and global effects that can be associated to the accumulation of sub‐lethal damage or oxidative stress in the sensitive volume of the cell. The latter is the volume where energy deposits can induce cell death, that is, the cell nucleus in this work.

We used an implementation of the NanOx biophysical model adapted to low‐energy ions to estimate biological quantities in the condition of TAT treatments. The main change is the consideration of the ion's energy loss in cell nuclei. More specifically, we took into account the variation of the density of local lethal and global events as a function of the ion's energy loss during the nucleus traversal. This low‐energy implementation relies on two approximations: narrow radiation tracks and the establishment of charged‐particle equilibrium. First, the secondary electrons have a low enough energy to stay within the ion's track (region defined by a parallelepiped of 200 nm side around the ion's track^35^). Second, the energy deposited by an ion track in a volume is equal to the difference between its kinetic energy at the entrance ($E_{i}$) and at the exit ($E_{f}$) of the volume. More details about the low‐energy implementation of the NanOx model are given in the paper dedicated to it.^36^ It is worth noting that the NanOx biophysical model is a coherent framework developed to be consistent between energy ranges from low‐energy targeted therapies to hadrontherapy.

The NanOx biophysical model and the Geant4 MC simulations do not interact directly with each other. A dedicated application was developed to combine outputs from both models: NanOx mono‐energetic radiobiological outputs as a function of the kinetic energy of the ion and the cell line considered, and CPOP‐Geant4 kinetic energies of ions entering ($E_{i}$) and exiting ($E_{f}$) cell nuclei of the spheroid registered in each irradiation configuration.

This coupled implementation allowed us to estimate the number of local lethal and global events for each couple ($E_{i}$, $E_{f}$) calculated from the Geant4 MC simulations. These events are summed over all the alpha tracks encountering nuclei. In consequence, for a given multi‐cellular irradiation configuration, the survival probability, S, of a cell was calculated with the expression:

(2) $$S=\exp(-\left(n_{L}+n_{G}\right))$$

with $n_{L}$ and $n_{G}$, the total number of local lethal and global events created by ions that hit the cell nucleus.

The corresponding TCP was calculated with a binomial law as follows:^37^

(3) $$\mathrm{TCP}=\prod_{i=1}^{n}\left(1-S_{i}\right)$$

where $S_{i}$ is the survival of the $i_{th}$ cell, and *n* is the number of “critical” cells, assumed to be equal to the total number of tumor cells. The mean TCP was obtained by averaging the calculated values over 20 to 500 irradiation configurations to get low statistical fluctuations.

The last biophysical index used in this study is the activity per cell (APC) required to get a TCP of 0.9 ($A_{TCP=0.9}$), as it was defined in the MIRD 22 Pamphlet^38^ for tumor control.

Cell survival probabilities were calculated for the HSG, V79, and CHO‐K1 cell lines. The corresponding reference parameters for a photon irradiation are given in Table 1. With the CPOP compaction algorithm, the cell nucleus cannot be deformed, but its radius can be reduced down to 80% of its maximum. In the cell survival calculations, the lethal function is normalized so that the number of nanotargets in the nucleus remains constant.^36^

**TABLE 1:** Geometrical and reference (i.e., for photon irradiation) biophysical parameters used to represent the different cell lines used in this study: HSG, V79, and CHO‐K1.

Cell line	$\alpha_{ref}\left(Gy^{-1}\right)$	$\beta_{ref}\left(Gy^{-2}\right)$	Nucleus radius (μm)	Cell radius (μm)
HSG	0.313	0.062	6.7	7.3
V79	0.184	0.020	5.2	7.1
CHO‐K1	0.228	0.020	3.85	7.0

The size difference between cell lines is taken into account in NanOx by changing the radius of the cylinder used to model cell nuclei and compute radiobiological outputs as a function of ion's kinetic energies. In CPOP, the 3D cells generated for the MC simulations have their nucleus radius and maximum cell radius modified to match the nucleus radius used in NanOx simulations.

The relative biological effectiveness (RBE) was defined as the dose ratio needed from a reference external photon beam irradiation to obtain the same mean survival as in a TAT irradiation, at the spheroid scale. This translates to the relation:

(4) $$\mathrm{RBE}=\frac{-\alpha_{ref}+\sqrt{\alpha_{ref}^{2}-4\cdot \beta_{ref}\cdot \ln\left(S\right)}}{2\cdot \beta_{ref}\cdot D}$$

where S is the average cell survival in a spheroid that absorbed the dose D. The reference irradiation coefficients chosen are $\alpha_{ref}=0.313$ ${\mathrm{Gy}}^{-1}$ and $\beta_{ref}=0.062$ ${\mathrm{Gy}}^{-2}$, issued from the work of Furusawa et al.,^39^ where HSG cells were irradiated by a 140 kV x‐ray machine. Comparisons were made with $\alpha_{ref}=0.259$ ${\mathrm{Gy}}^{-1}$ and $\beta_{ref}=0.040$ ${\mathrm{Gy}}^{-2}$ using 4 MeV LINAC x‐ray machines.^40^

To summarize, the cell survival probability (S), the TCP, the APC required to get a TCP of 0.9 ($A_{TCP=0.9}$), and the RBE are the biophysical indexes considered in this work. The spheroid (considered as a cancer lesion in this work) dose to get a TCP of 0.9 ($D_{TCP=0.9}^{\text{spheroid}}\text{[Gy]}$) was also calculated.

### 2.3 Spheroid modeling

One of the important issues of MC modeling in TAT is the lack of biological data of intratumoral or intracellular radionuclide distributions that can be used as realistic input parameters for simulations. Injected activity of a radiopharmaceutical cannot be easily converted into a number of alpha particle emissions in the tumor. The targeting efficacy strongly depends on the targeting vector (e.g. antibody or peptide) and the tumor environment.^41^, ^42^

The alpha‐camera method^43^ allows to measure ex vivo activities of alpha particles in a tissue. This system was used, for example, in the in vivo experimental study of Chouin et al.,^30^ on mice with xenografted OVCAR‐3 tumors irradiated with 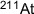. In this study, the estimated mean number of alpha decays per cell was 42 after a 400 kBq injected activity, assuming 74% of spheroid compaction with 95 μm radius. This number was quantified on a tissue with homogeneous distribution of radionuclides per cell. We used these values to design our TAT treatment initial conditions, in particular the ones used to obtain the results presented in Figures 3 and 4.

**FIGURE 2:**
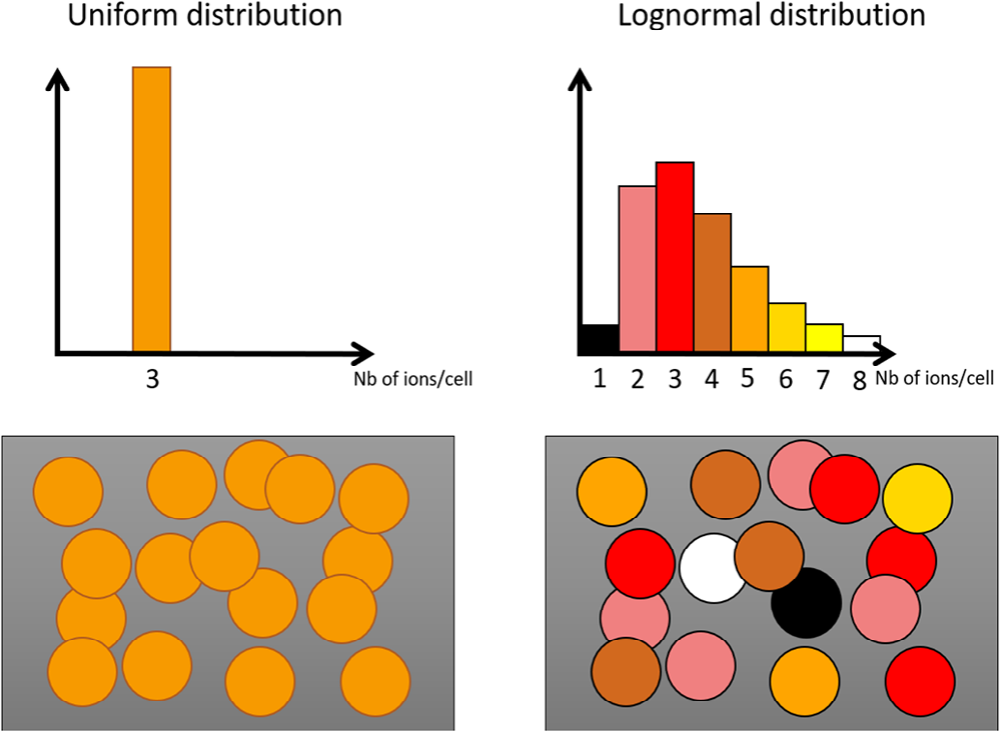
Schematic representation of a 2D section of a spheroid, each circle represents a cell containing a given number of ions; the distribution of ions per cell is shown in the top part of the figure. Left: uniform distribution with 3 ions per cell. Right: lognormal distribution with average of 3 ions per cell and shape factor of 0.5.

The spheroid construction is performed by generating cells within a defined spheroid radius, using initial spherical cell and nucleus shapes with maximum dimensions of a given cell type. When the cell density increases, CPOP allows to deform the cell membrane and cut the cytoplasm to avoid cell overlap. Nucleus size can also be reduced, without being deformed, until a minimum of 80% (default value) of its initial size, to allow higher spheroid compaction and faster spheroid construction convergence. An example of an OVCAR‐3 spheroid with 95 μm radius and 75% compaction (containing 3069 cells of maximum nucleus and cell radius of 5.5 and 6.9 μm, respectively) is shown in Figure 1.

### 2.4 Parametric study

To quantify the effect of potential intracellular radionuclide distributions (ICRD) that may be encountered according to the targeting vector used, tumor environment, or metabolism, four different distribution types inside cells were considered for alpha‐emitters: membrane, cytoplasm, cytoplasm + nucleus, and nucleus only.

**FIGURE 3:**
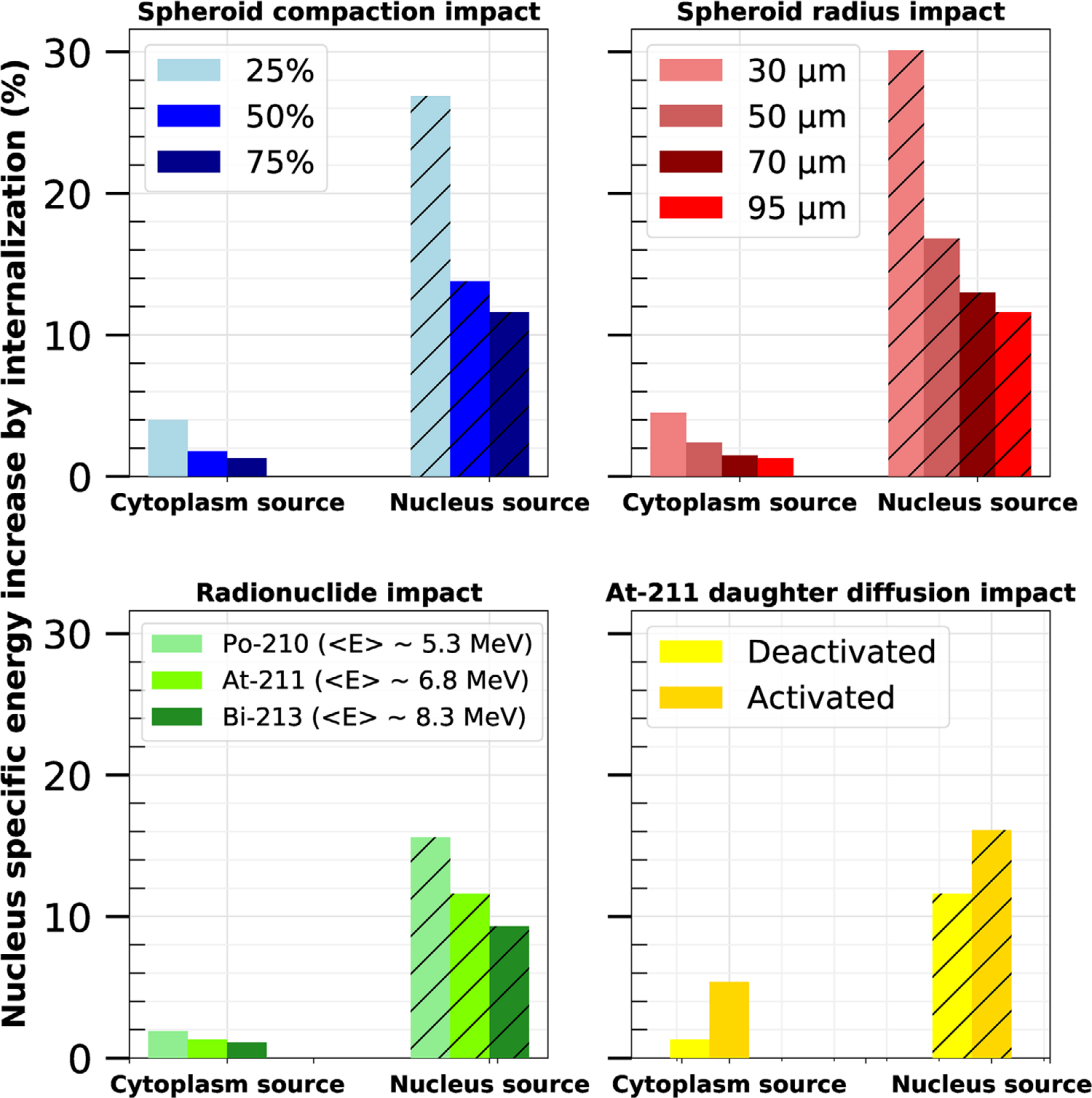
Mean nucleus specific energy increase with cytoplasm and nucleus radionuclide source distributions compared to the reference case of membrane distribution. Default parameters: 95 μm spheroid radius, 75% compaction, irradiated by 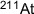. In each plot, one parameter was changed at a time.

**FIGURE 4:**
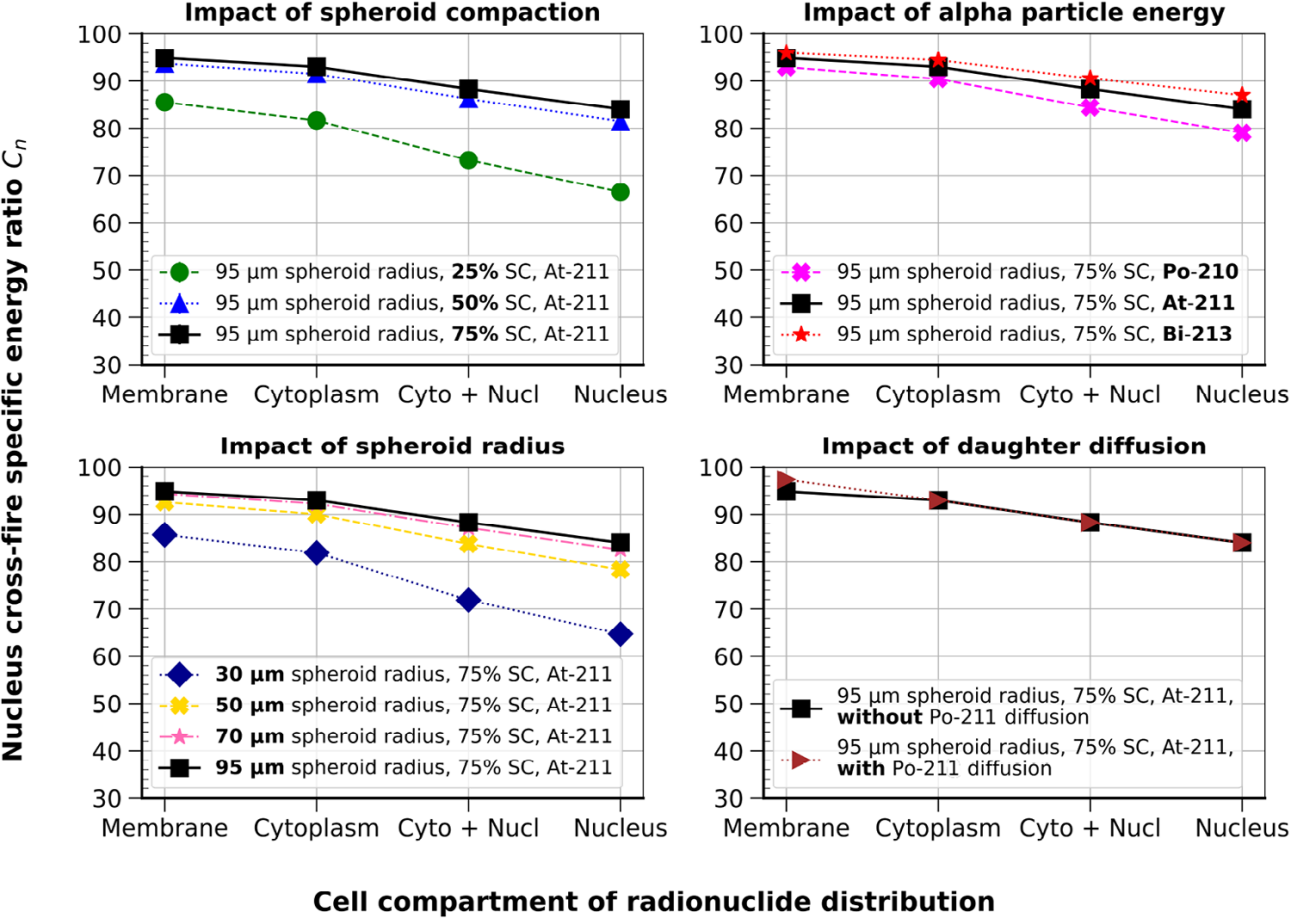
Average nucleus cross‐fire specific energy ratio $C_{n}$ calculated for each intracellular radionuclide distribution and set of influence parameters, for a fixed number of 42 alpha decays per cell. The top left plot shows the spheroid compaction (SC) impact study, the top right the alpha particle energy, the bottom left the spheroid radius, and the bottom right the radionuclide daughter diffusion study. The data in black solid lines were calculated with the default parameter conditions: 95 μm spheroid radius, 75% spheroid compaction, and 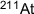 irradiation, without daughter diffusion. Lines between data points are plotted for visualization purposes. Statistical uncertainties are plotted at 2σ, but are small enough to be hidden by the symbols.

To obtain Figures 3 and 4, we used 42 alpha particles per cell^30^ (i.e., 1.12 mBq of 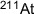 per cell), generated randomly in the chosen cell compartment. The default configuration consisted of a spheroid of 95 μm radius and 75% compaction treated with 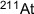. The OVCAR‐3 cell line geometry (maximum nucleus and cell radius of 5.5 and 6.9 μm, respectively)^44^ and the NanOx parameters of the HSG cell line were used by default.^21^ The geometry choice was made to correspond to the Chouin et al. measurements taken as reference. In the absence of radiobiological parameters for the OVCAR‐3 cell line, the HSG NanOx parameters were used to define a reference configuration, the impact of this choice is studied in the following. All treatment conditions were considered with the hypothesis that 100% of tumor cells were targeted by 42 radionuclides (no cold or hot zones in the spheroid), to reproduce the measurements of Chouin et al. This allows easier quantification of the impact of other parameters. From these conditions, a systematic parametric study was performed to quantify the impact of ICRD in various configurations, extending observations from the literature.

In the following simulations, both physical indexes (nucleus dose and cross‐fire contribution) as well as biological ones (TCP, RBE) were calculated.

Table 2 presents the reference configuration gathering the parameters of the reference irradiation and irradiated system.

**TABLE 2:** Reference parameters of the irradiated system and of the irradiation. ITA: Intratumoral activity; ICRD: intracellular radionuclide distribution.

Irradiated system							
Spheroid			Irradiation				
Radius	Compact.	Cell line	Radionuclide	Daughter diffusion	ITA distribution	ICRD	Decay per cell
95 μm	75%	HSG	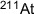	No	Uniform	Membrane	42

#### 2.4.1 Study with uniform intratumoral activity

##### 2.4.1.1 Impact of spheroid compaction and size

One of TAT's main clinical indications is the treatment of micrometastases, that may have multiple sizes. Using an analytical model, Goddu et al.^6^ found that the multi‐cellular cluster radius had an influence on the self‐dose to cross‐dose ratio only below 50 μm radius, with 74% cell compaction. It corresponds approximately to the range of the 5.3 MeV alpha particle emitted by 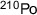, the radionuclide that they considered.

With CPOP, spheroid compactions of 25%, 50%, and 75%, and sizes from 30 to 95 μm radius were investigated and compared to a monocellular geometry irradiation. These choices were made to compare our results to existing literature^4^, ^6^, ^8^ and to go further in the range of potential variabilities encountered in clinical cases. Spheroids highly compacted up to 75% were used to reproduce as much as possible the compaction of tumors for in vivo or clinical cases. On the opposite, smaller compactions could represent in vitro spheroids,^20^ and smaller sizes are within the range of micrometastases.

##### 2.4.1.2 Impact of radionuclide choice

Various alpha‐emitters are investigated in TAT. To study the importance of alpha particle energy in our results, different radionuclides were considered. The one with the highest emitted energy is 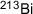, which 98% of the time emits an 8.4 MeV alpha particle (∼84 μm of path length). For the lowest encountered energy, 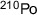 was used to maximize the possible intracellular distribution impact as it emits a 5.3 MeV alpha particle as a first decay (∼40 μm of path length). Its half‐life of 138 days does not make it a relevant candidate for therapeutic applications, but it has been used in in vitro studies.^45^ 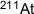 was used by default in our study, because of the intermediate energy of its alpha particle, which is on average 6.83 MeV (mean path length of 70 μm).

##### 2.4.1.3 Impact of daughter diffusion

For 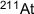, diffusion of its alpha‐emitting daughter, 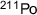, was considered. This process occurs 58% of the time with a physical half‐life of 0.5 s. As the chemical properties change with the radionuclide nature, 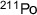 may not be fixed anymore to the vector and can diffuse before decay. A one‐dimensional thermal diffusion equation was considered following the method proposed by Palm et al.^16^ Two main hypotheses were made. First, radionuclides were unable to escape their primary cell compartments, an assumption made according to other studies.^46^ Second, to expand the diffusion model to multi‐cellular clusters, free radionuclides were allowed to diffuse in the inter‐cellular matrix without being able to enter another cell. Results were compared with the monocellular ones of Palm et al.^16^

##### 2.4.1.4 Impact of activity per cell

The APC was reduced from 1.12 to 0.027 mBq (i.e., a number of alpha particles per cell varying from 42 to 1) to study its influence on $Z_{n}$ and TCP calculations. The impact of the cell line and the fluctuations of radionuclides per cell were also studied as a function of APC. Activity calculations were performed assuming an instantaneous decay. The choice of using APC instead of number of primary particles per cell was made for simpler comparison to the literature (e.g., cell survival measurements as a function of APC of Neti et al. 2007,^45^ or the Chouin et al. 2012^30^ number of decays per cell used in this study).

Moreover, we reproduced the multi‐cellular study proposed by Charlton et al.^8^ to compare our simulation framework predictions with the literature. Charlton et al. compared the mean cell survival in a multi‐cellular cluster with various intracellular radionuclide distributions. The spheroid cluster had a 100 μm radius, with 40% compaction, with non‐overlapping cells. The radionuclide injected was 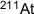, with four alpha particles per cell, giving a total spheroid dose of ∼1.5 Gy. The maximum cell radius modeled was 5.88 μm, and the maximum nucleus radius was 5.68 μm. Within cells, radionuclides were either attached to the membrane, or distributed in the cytoplasm or the nucleus. The study was performed with various cell lines, including the V79 cell line. For the cell survival calculations, we applied the low‐energy implementation of the NanOx model, as previously described, using the V79 cell line. In the work of Charlton et al., the cell survival calculations were based on the probability of lethal event creation in the cell nucleus. More specifically, the mean cell survival was computed by averaging the cell survival over a Poissonian distribution of alpha passages through the nucleus. For i alpha passages in the nucleus, the probability of cell survival was given by exp(−iT/λ), where T is the mean chord length in the nucleus and λ the mean free path associated with the lethal event creation. λ was experimentally tabulated as a function of the linear energy transfer (LET) of incident ions and the cell line considered.^47^ For LET below 100 keV/μm, they considered a fit of the mean free path between two lethal events, regardless of the cell line (tested for various cell lines, including V79). Above 200 keV/μm, they considered this mean free path constant.

#### 2.4.2 Impact of cell lines and fluctuations in ITA

##### 2.4.2.1 Impact of cell line with uniform ITA

To evaluate the robustness of this study against cell types, several cell lines were considered. We used the 3 cell lines for which NanOx radiobiological parameters were already available: HSG, V79, and CHO‐K1 (OVCAR‐3 cell line is not available). The corresponding maximum nucleus and cell radii are given in Table 1 for HSG,^48^, ^49^, ^50^ V79^51^ and CHO‐K1.^52^, ^53^, ^54^, ^55^, ^56^, ^57^ Spheroid's geometries as well as NanOx radiobiological coefficients were modified in our calculations accordingly. In line with a previous work,^36^ we assumed that the number of nanotargets in the nucleus of a given cell line is constant, whatever the nucleus shape and size. Therefore, the lethal functions used in this study were taken from an earlier study,^58^ except for a normalization factor accounting for the change of nanotarget density. It is worth noting that this specific study on cell lines combines both the impact of different ratios of nucleus and cell volumes and different radiosensitivities to alpha particles.

##### 2.4.2.2 Impact of lognormal fluctuations in ITA

The number of alpha particles in each cell of the spheroid was distributed according to a lognormal law:

(5) $$\begin{array}{cc} f(A_{0}) & =\frac{1}{A_{0}\sigma \sqrt{2\pi }}\exp\left(-\frac{1}{2}\frac{{\left(\ln\left(A_{0}\right)-\langle \ln(A_{0})\rangle \right)}^{2}}{\sigma^{2}}\right), \end{array}$$

(6) $$\begin{array}{cc} \text{with}\;\langle \ln(A_{0})\rangle & =\ln\left(\langle A_{0}\rangle \right)+\frac{\sigma^{2}}{2} \end{array}$$

$A_{0}$ is the mean activity in a cell (later converted into number of alpha particles), $f(A_{0})$ the associated probability density function, and σ is the shape factor of the distribution. This aims to reproduce experimental conditions where this type of distribution has been observed.^11^, ^12^, ^13^, ^14^ The shape parameter σ was set to 2.0, to best fit the experimental cell survival values of Neti et al.,^45^ at 100% cell labeling.

Figure 2 illustrates the number of ions generated for each cell in the case of a lognormal distribution. At the spheroid level, the average number of ions per cell is identical to that in a uniform distribution, but with fluctuations between cells.

## 3 RESULTS

### 3.1 Study with uniform intratumoral activity

#### 3.1.1 Impact of spheroid parameters, radionuclide choice, and daughter diffusion

Figure 3 presents the average nucleus specific energy ($Z_{n}$) increase with cytoplasm and nucleus ICRD, compared to the reference case of membrane distribution. Each subplot highlights the impact of a different irradiation parameter: spheroid compaction, spheroid radius, alpha particle energy spectra, and radionuclide daughter diffusion. Placing alpha‐emitters in the cytoplasm increased $Z_{n}$ at a maximum of 5% compared to the case of membrane distribution, regardless of the irradiation parameters.

In the default configuration, nucleus radionuclide distribution raised $Z_{n}$ by 11.6%. At maximum, it increased up to 27% and 30% in the 25% compaction and 30  μm radius spheroid conditions, respectively. In terms of alpha energy influence, the percentage of $Z_{n}$ increase with nucleus distribution, using 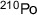, 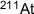, and 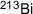 alpha energy spectra, was of 15.6%, 11.6%, and 9.3%, respectively. Finally, taking into account 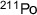 diffusion before decay increased the $Z_{n}$ ratio of 4.1% and 4.5% in the cytoplasm and nucleus internalization cases, respectively.

In the mono‐cellular case (OVCAR‐3 geometry), not shown in the figure, the rise of $Z_{n}$ was 214% and 296%, with cytoplasm and nucleus distributions, respectively, with respect to the case of membrane distribution.

The average ratio of cross‐fire nucleus specific energy $C_{n}$, is depicted for each radionuclide distribution and set of influence parameters in Figure 4. The value of $C_{n}$ ranged between 65% and 97%. In all cases, the closer the alphas were emitted from the nucleus, the lower the $C_{n}$. In the default irradiation case (black solid lines), the $C_{n}$ ranged between 84% to 95% for the nucleus to membrane‐only distributions, respectively. Decreasing the spheroid radius to 30 μm decreased the nucleus cross‐fire specific energy ratio to values ranging from 65% to 88% for nucleus‐to‐membrane source distributions, respectively. Values were similar for the 25% spheroid compaction. As for specific energy ratios, the alpha particle's energy and daughter radionuclide diffusion impacted only slightly the results, with, for example, 2.5% more cross‐fire contribution when diffusion was considered.

With 42 alpha decays per cell on average (1.12 mBq per cell), the biophysical calculations led to a TCP of 1 for all intracellular distributions and treatment conditions considered, due to the very high dose absorbed to the spheroid (> 28 Gy).

#### 3.1.2 Impact of activity

When APC varied from 0.027 to 1.12 mBq, the ratio of specific energy $Z_{n}$ with radionuclides distributed in the nucleus and the membrane was constant and equal to 1.116 ± 0.002, with the default irradiation parameters.

**FIGURE 5:**
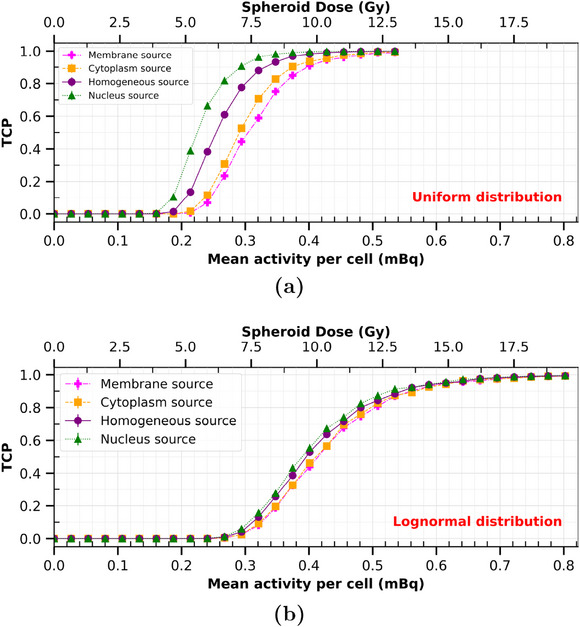
Tumor control probability (TCP) calculated in a 95 μm radius HSG spheroid of 75% compaction, treated with 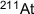, as a function of the mean activity per cell (APC) (lower abscissa), the spheroid dose (upper abscissa), and of radionuclide intracellular distributions. Lines between data points are plotted for visualization purposes. Error bars at ± 2σ are small enough to be contained within the data points. Plots (a) and (b) correspond to uniform and lognormal distributions of the radionuclide number per cell, respectively.

In Figure 5 Plot (a), the TCP calculations are presented for an activity of 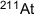 per cell varying from 0.027 to 0.534 mBq, for the four different ICRD considered. Above 0.534 mBq per cell, the TCP was always equal to one, as for 1.12 mBq (not shown in the figure). Below 0.534 mBq, the TCP was decreasing with a sigmoid tendency down to zero below 0.162 mBq per cell. The strongest impact of cell internalization can be observed between 0.22 and 0.35 mBq per cell. At maximum, the TCP was multiplied by 1.3 (at ∼0.27 mBq) between cytoplasm and membrane distributions, and by 9.5 (at ∼0.24 mBq) between nucleus and membrane distributions. The APC of alpha‐emitters required to get a TCP of 0.9 ($A_{TCP=0.9}$ 1) was 0.387, 0.363, 0.318, and 0.278 mBq for membrane, cytoplasm, homogeneous, and nucleus distributions, respectively. In other words, there is a relative $A_{TCP=0.9}$ decrease of 28% for nucleus distribution compared to the membrane only. Table 3 presents the spheroid dose (or lesion dose) required to get a TCP of 0.9, as a function of the intracellular radionuclide distribution for the reference irradiation configuration. These dose values were, for the uniform case, 9.35, 8.82, 7.71, and 6.77 Gy for membrane, cytoplasm, homogeneous, and nucleus distributions, respectively. Similarly to APC, there is a decrease of 28% spheroid dose required for tumor control between membrane and nucleus source distributions.

**TABLE 3:** Spheroid absorbed dose to obtain a TCP = 0.9, in the reference irradiation configuration, as a function of the intracellular radionuclide distribution and the fluctuations of intratumoral activity.

	$D_{TCP=0.9}^{\text{spheroid}}\text{(Gy)}$	
Intracellular source distribution	Uniform	Lognormal fluctuations
Membrane	9.35	13.08
Cytoplasm	8.82	12.99
Homogeneous	7.71	12.66
Nucleus	6.77	12.38

**FIGURE 6:**
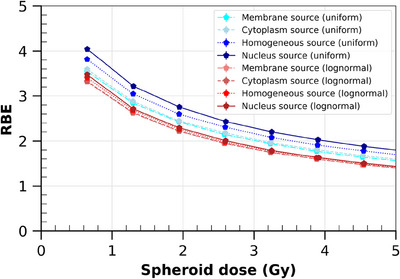
RBE as a function of the total absorbed dose in the spheroid (Gy) with the HSG cell line. The RBE was calculated for the four studied intracellular radionuclide distributions. Calculations were made for both uniform and lognormal distributions of radionuclides within cells. The reference photon coefficients were extracted from Furusawa et al.^39^ Error bars are plotted at ± 2σ.

Figure 6 shows the RBE calculations performed as a function of the whole absorbed dose in the spheroid, with different ICRD with the HSG cell line. The spheroid dose is directly correlated to the APC. Values of RBE ranged from 1.4 to 4.3 when the spheroid dose varied from 5 to 0.6 Gy, respectively. For an averaged 10% cell survival (∼1.3 Gy) and nucleus distribution, RBE was found approximately equal to 3.3 with the Furusawa et al. reference photon irradiation^39^ and 4.0 for the one from Aoki‐Nakano et al.^40^ (for a uniform ITA distribution). The impact of the ICRD on RBE varied only slightly with the spheroid dose. When radionuclides were emitted from the nucleus instead of the membrane, there was a 13% to 17% increase in the RBE within the considered dose range.

In the comparison of mean cell survival in the simulation setup of the Charlton et al. study^8^ (100 μm radius spheroid with 40% compaction and four 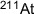 decays in each cell), the three ICRD considered were: membrane, cytoplasm, and nucleus. For these three distributions, the mean cell survivals were, respectively, 6.3%, 6.4%, and 6.1%. With our modeling, these were 2.4%, 2.0%, and 0.25%.

### 3.2 Impact of cell lines and activity fluctuations

#### 3.2.1 Impact of the cell line with uniform ITA

When cell geometry and radiobiological coefficients were changed to fit the HSG, V79, and CHO‐K1 cell lines, $A_{TCP=0.9}$, were respectively equal to 0.407, 0.210, and 0.270 mBq for the membrane distribution, and to 0.296, 0.150 and 0.176 mBq for the nucleus distribution. The ICRD has the strongest impact on the CHO‐K1 cell line, with a relative decrease in $A_{TCP=0.9}$ of membrane‐to‐nucleus distributions of about 38%, while it is about 28% for the other 3 cell types. Results for all configurations are summarized in Table 4

**TABLE 4:** $A_{TCP=0.9}$ values (mBq) with the reference configuration considered in this work (OVCAR‐3 geometry and HSG radiobiological coefficients, with uniform distribution of radionuclides), compared to values obtained with the geometry and radiobiological coefficients of the HSG, V79, and CHO‐K1 cell lines. The absolute values are given for the configuration OVCAR‐3 geometry and HSG cell line. For the other configurations, relative differences with the reference values are given. Simulations were performed to obtain a mean error of ± 0.001.

Configuration					
Geometry	Coefficients	Membrane	Cytoplasm	Homogeneous	Nucleus
OVCAR‐3	HSG	0.387	0.363	0.318	0.278
HSG	HSG	+5%	+7%	+5%	+7%
V79	V79	−46%	−43%	−43%	−46%
CHO‐K1	CHO‐K1	−30%	−31%	−31%	−37%

#### 3.2.2 Impact of activity with lognormal fluctuations of ITA

With the chosen default irradiation parameters, switching from a uniform to a lognormal ITA distribution per cell increased $A_{TCP=0.9}$ values to 0.541, 0.536, 0.521, and 0.509 mBq for the membrane, cytoplasm, homogeneous, and nucleus distributions, respectively (Figure 5 Plot (b)). This highlights that the lognormal intratumoral distribution reduces the impact of intracellular distribution. The maximum TCP difference is found at 0.348 mBq, with 1.5‐fold higher TCP between the membrane and nucleus distributions. In terms of $D_{TCP=0.9}^{\text{spheroid}}\text{(Gy)}$ (Table 3), applying lognormal fluctuations to the radionuclide distribution per cell reduces the impact of intracellular source distribution from −28% (uniform case) to −5% from nucleus to membrane distributions.

Similarly, a lognormal ITA distribution instead of a uniform one decreases the RBE, as observed in Figure 6. With the nucleus distribution, there is between a 14% and 24% decrease in RBE. For the membrane distribution, the differences vary between 6% and 14%. The impact of ICRD on RBE is also strongly reduced compared to a uniform ITA distribution, with differences of 4.8% to 0.8% between membrane and nucleus distributions.

## 4 DISCUSSION

This study aimed to extensively assess the variations of physical and biological indexes as a function of intracellular radionuclide distributions in TAT. In the literature, Goddu et al.^6^ calculated the cross and self‐dose absorbed in multi‐cellular geometries using different energies and particles, including alpha‐emitters, using an analytical model. They found that cross‐dose was the major contribution to the total dose. Therefore, the contribution of ICRD has less impact than that observed in monocellular studies. Another study conducted by Charlton et al.^8^ used an in‐house MC model in multi‐cellular geometries to evaluate the effect of ICRD as well as tumor compaction and size on cell survival. The biological effect was determined through a LET‐depending model.^7^ These two articles emphasized the need for further studies of multi‐cellular models to better understand these effects in TAT. This was the purpose of the present paper, proposing a systematic study of the influence of ICRD as a function of various irradiation and geometric parameters: spheroid compaction and size, alpha particle energy, radionuclide daughter diffusion, and fluctuations of ITA.

### 4.1 Study with uniform intratumoral activity

#### 4.1.1 Impact of ICRD on mean nucleus specific energy

Overall, the impact of ICRD was relatively low on physical indexes as the mean nucleus specific energy. Changing from a membrane distribution to a cytoplasmic (resp. nuclear) distribution led to a maximum increase of the mean nucleus specific energy of 5% (resp. 30%). This result can be explained by the long range of alpha particles in TAT relative to cell size (ranges typically from 3 to 5 cell diameters) and most of the deposited energy is due to cross‐fire, in agreement with the conclusions of Goddu et al.^6^, ^8^ Not surprisingly, the impact of intracellular radionuclide distribution is lower in spheroid than in a monocellular model.^4^

#### 4.1.2 Impact of spheroid size and compaction

The largest impact of ICRD on mean nucleus specific energy was found for the smallest spheroid sizes and the lowest compaction, mainly due to a reduction of cross‐fire contribution. Hence, for such small micrometastases, the use of targeting molecules allowing internalization of the radionuclides into the cell's cytoplasm or nucleus could be of importance.

#### 4.1.3 Impact of alpha particle energy

At the scale of a 95 μm radius spheroid, changing the energy spectra of alpha particles only modified the dose increase by a few percent. The range differences were minor compared to the size of the spheroid. The emission energy of alpha particles had almost no impact on the mean specific energy, no matter the chosen radionuclide.

#### 4.1.4 Impact of radionuclide daughter diffusion

When diffusion of 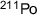 was considered in our multi‐cellular model, the mean specific energy to the cell nucleus did not vary as much as what was obtained with the monocellular model of Palm et al.^16^ They found that the total dose to the cell nucleus was divided by 2 when 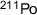 diffused away from the original binding site, with its half‐life of 0.5 s. In a multi‐cellular model, the daughter diffusion only increases the cross‐fire effect, as long as the alpha particle range is not too long compared to the size of the tumor, and the daughter half‐life is short. In this work, the average dose to cell nuclei was divided by 1.04 when considering 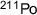 diffusion, which can therefore be considered negligible.

#### 4.1.5 Discussion about other radionuclides

Other radionuclides with more complex decay chains, like Ac‐225, have daughters with half‐lives of several minutes. These radionuclides can diffuse away from the tumor and irradiate critical organs, which changes the biological effect.^59^ Different encapsulation techniques are studied to mitigate this diffusion effect.^60^, ^61^ Dedicated modeling for the diffusion of these radionuclides would need more complex integration with global metabolic considerations, that goes beyond the scope of this study.

#### 4.1.6 Impact of activity on RBE and TCP

The effects of intracellular distribution on biological outcomes could not be highlighted using the Chouin et al. irradiation conditions. Indeed, with the 100% cell labeling hypothesis, all cells were dying in every situation with such a high value of 42 alpha particles per cell, since only a few (from about 1 to 20) alpha particles may be sufficient to kill a cell.^38^, ^62^ However, when the APC was changed, a threshold area was observed where the intracellular distribution had a major effect on TCP, up to a factor of 9.5 in extreme internalization and uniform intratumoral conditions. In the case of homogeneous irradiations (constant APC over the spheroid) with low APC, internalization into the cytoplasm and/or the nucleus can be critical.

Careful consideration must be given to TCP calculations at high dose. Currently, biophysical models are constrained by limited experimental data on cell survival, which are typically available only for dose values up to a few Gy. In this work, we assumed that cell survival in response to ion irradiation maintains the same linear‐quadratic trend at higher dose values as the one observed at low dose.

Finally, our RBE calculations represent the capability of irradiation to convert a physical dose to a spheroid into a biological effect. Mathematically, RBE is maximum when the dose is minimum. Please note, however, that we used external photon beams as the reference photon irradiation. Using photon irradiation with similar dose rates as those encountered in radionuclide therapy may impact this tendency. Due to the lack of such information for the chosen cell lines, radiobiological parameters obtained from classical cell irradiation experiments with external photons were used instead as reference. However, even the external photon reference chosen for the calculations (Furusawa et al.^39^ vs. Aoki‐Nakano et al.^40^ for the HSG cell line) induces a variation of approximately 25% in RBE absolute values, highlighting that the choice of the reference may be critical. With this in mind, we found that the RBE increased slightly when the radionuclides were distributed within the nucleus, compared to the membrane‐only distribution. This result is consistent with the literature^63^ and the TCP trend. However, we found that, no matter the absorbed dose to the spheroid, the RBE differences between the intracellular distributions were almost constant.

Some conclusions can be drawn from the estimate of RBE. First, the high value of RBE at 10% survival between 2.3 and 3.3 underlines the necessity to take properly into account the biological effect of alpha particles in TAT treatment planning systems. To compare with another treatment type, the RBE of protons in hadrontherapy has been considered constant and equal to 1.1 in some debated conventions.^64^ Despite this small value, it is still necessary to consider it properly in biological dose calculation so as not to induce overdosage in tissues, when prescribing the dose to the tumor. Considering the biological effect of alpha particles may be critical in TAT if one aims to prescribe the injected activities for personalized dosimetry. Second, the RBE cannot be set constant and would need to be linked to the radiopharmaceutical organ uptake quantification based on imaging.

The lesion dose required to get a certain TCP is an index used in the literature to compare clinical data between them. For example, Minguez et al.^65^, ^66^ calculated $D_{TCP=0.9}^{\text{lesion}}\text{(Gy)}$ with the Roeske and Stinchcomb formalism^67^ and the MIRD method,^68^ to compare with clinical treatments of metastatic castration‐resistant prostate cancer. They used 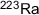 and 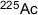 radionuclides. Depending on the parameters considered (mass density, cell radiosensitivity, nucleus radius, and lesion volume), they found $D_{TCP=0.9}^{\text{lesion}}$ values between 6.7 Gy (for a tumor volume of 1 ${\mathrm{cm}}^{3}$) and 116.5 Gy (for a tumor volume of 100 ${\mathrm{cm}}^{3}$). The lower the tumor volume, the lower the $D_{TCP=0.9}^{\text{lesion}}$. Due to the difference in the parameter values used in our work, direct comparison cannot be performed, but the order of magnitude is similar. At the time of the current manuscript, clinical data with 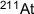 are scarce, but our modeling results considering various irradiation configurations might allow comparison with clinical data in the future.

#### 4.1.7 Impact of ICRD on mean cell survival: comparison with the study of Charlton et al

We found two major differences between the mean cell survival obtained by Charlton et al. and our calculations in spheroid clusters of the V79 cell line. Not only the variations of the mean cell survival were different as a function of the radionuclide distributions, but also the absolute values differed. For the membrane, cytoplasm, and nucleus distributions, the mean cell survivals were, respectively, 6.3%, 6.4%, and 6.1% for Charlton et al. and 2.4%, 2.0%, and 0.25% in our calculations.

These differences in cell survival predictions can be explained by the following differences between the two studies:

1. The parametrization of the models was performed with different experimental data.
2. The spheroid modeling with 40% compaction was performed without cells overlapping by Charlton et al., while the CPOP code used in our study allows for cell deformation. Hence, the nucleus volume represented on average 50% of the cell volume in our study and was fixed to 90% in the one of Charlton et al.
3. The model proposed by Charlton et al. took into account the radionuclide distributions only through the mean number of alpha passages in the nucleus. On the contrary, in our calculations, the slowing down of the alpha particles along their path and the associated increase of LET were also modeled.

Concerning the strong impact of radionuclide distributions on absolute cell survival in our study (from cytoplasm to nucleus distributions), it may be caused by the two latter differences. Regarding the spheroid modeling with CPOP, as the nucleus volume is smaller, when the radionuclide is located at the membrane, the probability of energy deposition in the nucleus is lower. Therefore, changing the radionuclide distribution has a larger impact on the CPOP modeling of the spheroid. However, this ballistic aspect cannot explain all the differences. Indeed, the dosimetry study showed that the dose deposition in the nucleus was only increased by about 10% when moving from the membrane distribution to the nucleus distribution. As a rough approximation, assuming a linear model of cell survival ($S=\exp(-\alpha Z_{n})$), we may translate the 10% dose increase into a power of 1.1 of the cell survival. This impact is far too low to explain the decrease of cell survival predicted in our study by a factor of 10 between the membrane and nucleus radionuclide distributions. In other words, the main difference between the two studies may be attributed to the consideration of biological effectiveness variation of alpha particles along their path.

### 4.2 Impact of cell lines and activity fluctuations

#### 4.2.1 Impact of cell lines on TCP

By fixing the number of nanotargets in the cell nucleus but changing the cell geometry from OVCAR‐3 to HSG (having the larger cell nucleus radius), we may expect two effects on $A_{TCP=0.9}$. On the one hand, increasing the nucleus radius increases the probability of nucleus impact, which should decrease cell survival. On the other hand, this increase in nucleus size leads to a reduction of the nano‐target density and hence to an increase in cell survival. As $A_{TCP=0.9}$ increased by about 6% from OVCAR‐3 to HSG geometries, whatever the radionuclide distribution, we can conclude that the reduction of the nanotarget density is the dominant effect.

Comparing the 3 cell lines (HSG, V79, and CHO‐K1), this effect was confirmed as reducing the radius of the cell nucleus to the ones of V79 and CHO‐K1, led to lower $A_{TCP=0.9}$ values. This is counterbalanced by the intrinsic radiobiological parameters of the cells, which ultimately leads to a higher radiosensitivity of the V79 line compared to CHO‐K1, despite the latter have the smallest nucleus. Finally, when looking at the relative variation of TCP values ​​as a function of ICRD, we showed that the ratio of the nucleus to cell volume of a cell line is the predominant parameter impacting the potential treatment efficacy of a radiopharmaceutical capable of internalizing the compartments, since the CHO‐K1 line is the one with the largest aspect ratio and showing the strongest TCP variation.

#### 4.2.2 Qualitative impact of activity fluctuations

The previously discussed results were obtained with a constant number of radionuclides per cell over the whole spheroid. When fluctuations of this number were included following lognormal distributions, cell survival probabilities (S), TCP, and RBE were reduced. This result is consistent with literature^13^ and could be interpreted by the overkilling effect, well‐known in hadrontherapy.^69^ Indeed, since the cell survival probability follows a decreasing exponential law with activity, the mean cell survival over a distribution of activity is larger than the cell survival to the average activity. The term “overkilling” corresponds to the fact that for the largest activities, the survival probability is close to zero, whatever the activity.

Another important result was that the impact of ICRD on TCP and RBE almost vanished with activity fluctuations. Regarding TCP, for constant total activity over the spheroid, the impact of ICRD appears in a small range of mean APC. In this range, applying lognormal activity fluctuations distributes the activity around the mean value, and therefore in and out of the aforementioned window. This convolution effect enlarges the window and reduces the sensitivity to intracellular radionuclide distribution. Similarly, as RBE depends on the mean cell survival, areas with low activity induce higher mean survivals for the same average dose compared to no activity fluctuations.

#### 4.2.3 Limitations and perspectives

The $A_{TCP=0.9}$ values could be sorted in the following order: V79 < CHO‐K1 < HSG, meaning that the V79 cell line would be more sensitive to alpha irradiation than the HSG one. However, one may notice that our chosen cell lines had similar cell radius dimensions. Considering larger cells of dimensions comparable to the range of alpha particles (e.g., giant cancer cells^70^) could change to a major extent the cross‐fire effect and the consequent impact of intracellular distribution. In addition, the 3 cell lines currently validated in the NanOx model are healthy cells (however, immortalized). Cancerous cells could react differently to alpha particle irradiations.^71^, ^72^ Nevertheless, this study gives boundaries on the possible impact of the cell line choice considered in predictive models, and shows that uncertainties due to such parameters may be rather small in comparison to the ones coming from intratumoral distributions.

One limitation of our study is that, like other biophysical models of RBE,^73^, ^74^ the NanOx model assumes that the cell nucleus is the only sensitive volume able to induce cell death. Our team is working on including a second sensitive volume corresponding to the extra‐nuclear region. The NanOx formalism has been generalized in that perspective.^36^ We also expect to extend this work to applications of boron neutron capture therapy (BNCT), where the range of alpha particles and lithium ions following a neutron capture is much smaller (below the dimensions of a single cell) than in TAT. In such conditions, a major impact of boron internalization would be expected, and the consideration of extra‐nuclear damage in the process of cell death would be even more interesting. This application would be possible thanks to the consistency of the NanOx model throughout a wide range of energies.^36^ The modeling could also be extended by using MC simulations to compute DNA damage, in addition to specific energy in nanotargets, using, for example, Geant4‐DNA physics and DNA geometries.^75^, ^76^, ^77^ This would allow, for example, the inclusion of a temporal dimension in predictions by considering the dynamic processes of DNA damage and repair. Besides, complex heterogeneous fixation schemes in the tumor are found in vivo.^30^, ^78^ The impact of these tumoral fluctuations on biological indexes will be explored in detail in the future. To validate more thoroughly such TAT modeling, further studies are planned to produce experimental data with a precise control of in vitro cell irradiation (with the description of sub‐cellular radionuclide distributions), and of in vivo based data with information on intratumoral radionuclide distribution.

## 5 CONCLUSIONS

The presented study aimed to offer some directives for assessing the potential errors that might occur in biological response predictions in TAT, based on the accumulated activity in tumors, for various irradiation configurations. Moreover, this is an example application of the low‐energy implementation of the NanOx model. Absorbed dose and TCP were computed with a combination of sophisticated MC and biophysical models, evaluating especially the impact of different intracellular radionuclide distributions. We found that precise modeling of such distributions was required in the case of isolated cancerous cells, small micro‐metastases, or tumors presenting regions with relatively low radionuclide concentration (i.e. a few radionuclides per cell), effects that have been highlighted thanks to the RBE and TCP biophysical calculations. In contrast, the distribution of radionuclides played a minor role with spheroids larger than the range of alpha particles and for activities higher than 0.40 mBq (equivalent to 15 alpha decays) per cell. We found, however, that fluctuations in the intratumoral radionuclide distribution, modeled with lognormal‐like distributions, dramatically impacted the TCP predictions and should thus be considered in TAT personalized treatment dosimetry protocols.

## CONFLICT OF INTEREST STATEMENT

The authors have no conflicts to disclose.

## Footnotes

1 Errors at 2σ were below 0.002.
